# A Rare Cause of Intestinal Obstruction: Invasive Lobular Breast Carcinoma Metastasizing to the Ileocecal Valve

**DOI:** 10.7759/cureus.90985

**Published:** 2025-08-25

**Authors:** Charalampos-Christos Douligeris, Eleni Boptsi, Charalampos Theocharopoulos, Dimitra Foteinou, Antonios Batis, Pavlos Lampropoulos, Othon Manolakos

**Affiliations:** 1 Department of Surgery, Laiko General Hospital, Athens, GRC; 2 Department of Surgery, Metaxa Cancer Hospital, Piraeus, GRC; 3 Department of Surgery, University of Colorado Anschutz Medical Campus, Aurora, USA; 4 Department of Medicine, National and Kapodistrian University of Athens, Athens, GRC

**Keywords:** breast cancer, emergency surgery, intestinal metastasis, intestinal obstruction, invasive lobular carcinoma

## Abstract

Invasive lobular carcinoma (ILC) has a higher propensity for gastrointestinal metastases compared to invasive ductal carcinoma (IDC). We present the case of a 65-year-old woman with metastatic ILC who developed intestinal obstruction due to ileocecal metastases 30 months after undergoing total mastectomy and adjuvant therapy for left-sided breast cancer (BC). Abdominal computed tomography (CT) demonstrated a transition point at the ileocecal valve. Surgical resection was performed to relieve the small bowel obstruction, and histopathology confirmed metastatic ILC with receptor discordance compared to the primary tumor. This case highlights the diagnostic and therapeutic challenges of intestinal metastases from BC, including receptor conversion and resistance to therapy. Molecular profiling and tailored treatment are crucial for optimal management of complex metastatic disease.

## Introduction

Breast cancer (BC) is the most common malignancy in women worldwide and, despite advances in prevention, screening, and treatment, it remains the second leading cause of cancer-related death in this population [1]. Invasive ductal carcinoma (IDC) is the most common histological subtype, followed by invasive lobular carcinoma (ILC), accounting for 70-80% and 10-15% of cases, respectively [2]. ILC arises from malignant cells within the lobules of the mammary glands and is distinguished by its slow, insidious pattern of spread.

BC exhibits subtype-dependent metastasis organotropism, which is regulated by an intricate crosstalk between cancer cells and the host organ microenvironment [3]. IDC shows a preference for parenchymal organs and the central nervous system, while ILC exhibits three- and fourfold higher propensity for distant spread to the ovaries and the gastrointestinal tract, respectively, compared to IDC [4]. The estimated rate of ILC metastasis to the GI tract varies significantly from 0.3% to 18% in autopsy series, with the stomach being the most common site. Intestinal metastases are a rare phenomenon and equally involve the large and small intestine [5].

In cases of intestinal involvement by BC metastasis, a major diagnostic challenge lies in differentiating metastatic disease from a primary intestinal malignancy, as both can present with abdominal pain, anemia, bleeding, or obstructive symptoms, making symptom-based differentiation not definitive. According to a systematic review of 96 patients with intestinal metastasis from BC, the most common presenting symptom was intestinal obstruction (40.6%), followed by abdominal pain (20.8%), rectal bleeding (10.4%), while in 12.5% of cases, the lesions were asymptomatic and detected incidentally [5]. Primary CRC presents primarily with altered bowel habits, abdominal pain, rectal bleeding, and anemia [6,7]. Given this overlap, histopathological and immunohistochemical evaluation remain essential for establishing the correct diagnosis.

At present, there are no definite guidelines for the management of BC metastases to the GI tract. Current evidence on surgical resection for isolated metastases in well-selected patients suggests a potential survival benefit. However, intestinal metastases most commonly occur in the setting of multiorgan spread, where systemic pharmacotherapy is used and surgery is typically reserved for intestinal complications. In the present study, we present a case of a 65-year-old woman with a history of ILC and multiorgan metastatic spread who presented to the emergency department with signs and symptoms of intestinal obstruction as a complication of metastatic ILC.

## Case presentation

**Figure 1:**
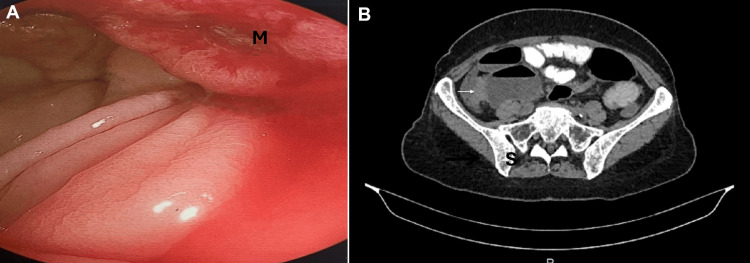
Endoscopic and imaging findings (A) Endoscopy shows a slowly oozing, ulcerated lesion representing intestinal wall infiltration by metastatic breast cancer. M: intestinal breast cancer metastasis; I: normal intestinal mucosa. (B) CT scan of abdomen and pelvis with oral and IV contrast showing a space-occupying lesion obstructing the ileocecal valve. Black arrow: ileocecal valve obstruction. S: dilated proximal small intestine loops

A 65-year-old woman with a history of stage IV, left-sided BC presented to the emergency department with complaints of right lower quadrant abdominal pain, vomiting, and abdominal distension, which had begun 12 hours prior to presentation. The patient had been diagnosed with a grade II, estrogen receptor (ER)-positive, progesterone receptor (PR)-positive, human epidermal growth factor receptor 2 (HER2)-negative ILC three years prior to presentation. Due to locally advanced disease involving the ipsilateral axillary and internal mammary lymph nodes at the time of diagnosis, the patient was offered neoadjuvant chemotherapy consisting of four cycles of epirubicin plus cyclophosphamide followed by five cycles of paclitaxel plus cyclophosphamide. Subsequently, the patient underwent a modified radical mastectomy, following a frozen section evaluation of a sentinel lymph node biopsy that was positive for malignant infiltration; histological examination of the specimen revealed yT2N2 disease. The patient received adjuvant radiotherapy and was commenced on hormonal therapy with letrozole. Six months after surgery, she was diagnosed with histologically confirmed metastasis to the bone marrow, and her treatment regimen was enhanced with capecitabine. Two years later, disease progression was detected on follow-up positron emission tomography (PET) scan, which revealed further bone metastases. Additionally, a gastrointestinal endoscopy was performed due to abdominal pain and revealed a slowly oozing, ulcerated lesion at the ileocecal valve, and a biopsy obtained during this procedure confirmed the diagnosis of metastatic ILC (Figure 1A), despite no evidence of intestinal involvement on the PET scan. Accordingly, her treatment was switched to the CDK4/6 inhibitor ribociclib, along with letrozole.

On presentation, the patient reported symptoms indicative of complete bowel obstruction, including abdominal distension, nausea, vomiting, along with a complete cessation of flatus and stool passage. A computed tomography (CT) scan of the abdomen and pelvis was performed, which revealed concentric wall thickening of the ileocecal junction, measuring approximately 2.8 cm in diameter, with no clear visualization of the lumen. This finding was attributed to a metastatic lesion causing intestinal obstruction at the ileocecal valve (Figure 1B). Tumor marker assessment revealed carcinoembryonic antigen (CEA), 4.3 ng/mL (reference: 0-5 ng/mL); CA 125, 68.2 U/mL (reference: 0-35 U/mL); and CA 15-3, 60.2 U/mL (reference: 0-30 U/mL).

Based on evidence of complete luminal obstruction on the CT, the patient underwent expedited exploratory laparotomy, which revealed peritoneal carcinomatosis and a mass involving the terminal ileum and right colon. Due to luminal obstruction, a right hemicolectomy was performed. Although a right hemicolectomy does not confer a survival benefit in the context of polymetastatic disease, where surgery is purely palliative, the macroscopically concerning appearance of the colon prompted a right hemicolectomy over a limited or atypical resection. The ileocecal valve was obstructed by an ulcerative lesion with a maximum diameter of 3.5 cm. The patient had an uneventful recovery and was discharged on postoperative day five. One month later, the patient restarted systemic treatment with paclitaxel and bevacizumab. She remains under close follow-up, with stable disease ten months after surgery.

**Figure 2:**
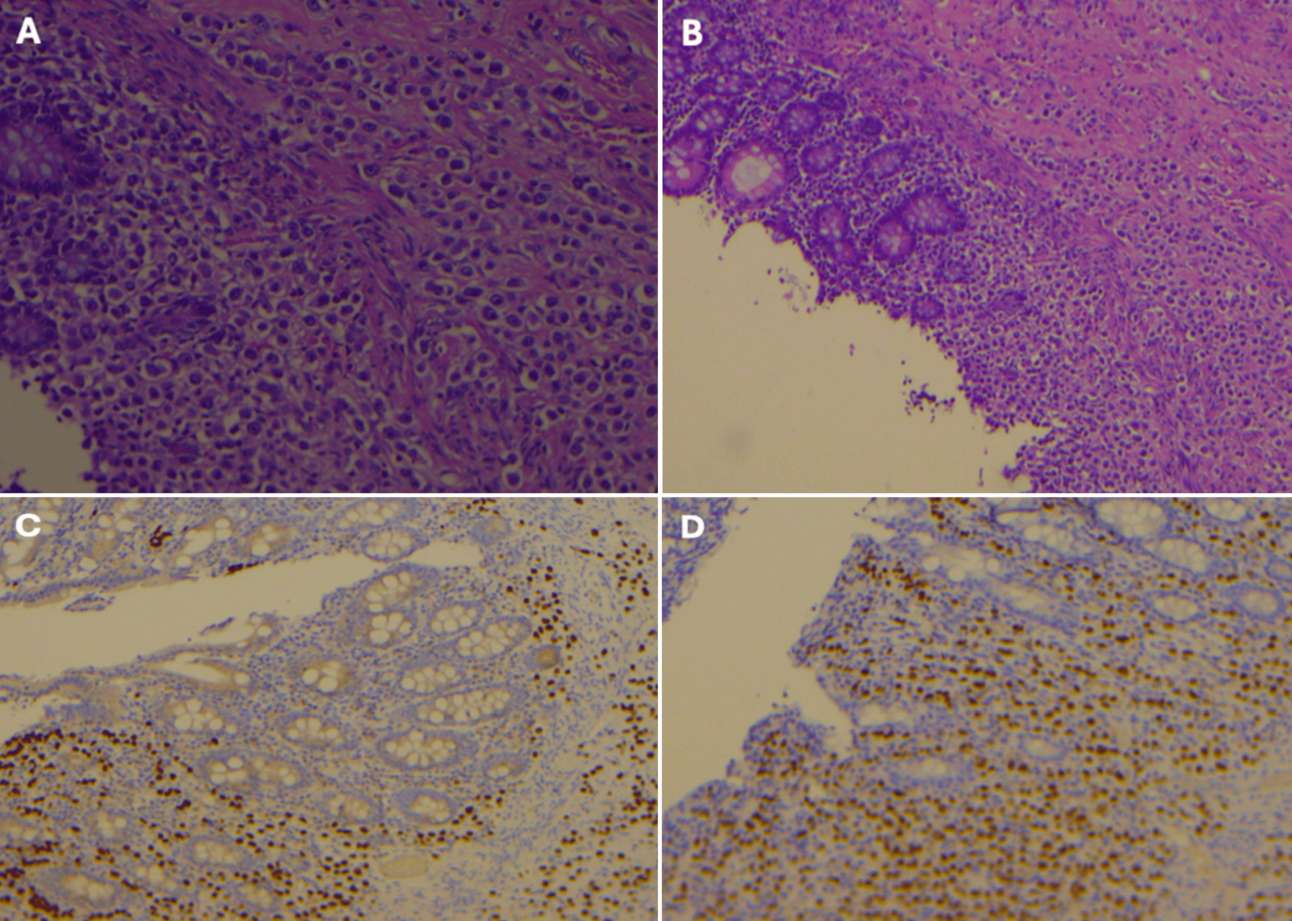
Histopathological and immunohistochemical evaluation of small intestinal metastases H&E: hematoxylin and eosin; IHC: immunohistochemistry; ER: estrogen receptor (A) H&E, x100: poorly differentiated carcinoma infiltrating the intestinal wall, composed of pleomorphic tumor cells with hyperchromatic nuclei and increased mitotic activity. (B) H&E, x100:  neoplastic infiltration extending beneath the mucosa, with partial preservation of glandular architecture. (C) IHC, x100: tumor cells positive for GATA3. (D) IHC, x100: tumor cells positive for ER

Histological examination of the surgical specimen revealed metastatic ILC ulcerating the mucosa. Metastases were also identified in at least three lymph nodes within the pericolic fat and in sections from the omental tissue. Immunohistochemical staining confirmed the BC origin, with the tumor testing positive for GATA3 and ER and negative for PR and HER2 (Figure 2).

## Discussion

Approximately 20-30% of patients with BC develop distant metastases, with ILC being more likely to metastasize than IDC [8,9]. While bones, liver, lung, and brain are the most common metastatic sites, different histological subtypes exhibit distinct organotropism, exhibiting different metastatic patterns [10,11]. Notably, ILC has a higher tendency to metastasize to the gastrointestinal tract compared to the ductal type [11]. 

Tissue-specific gene signatures and signaling pathways underlying organ-specific metastasis have been identified by comparing tumor cells from the primary BC with those from distant metastases [5]. These findings provide insight into the mechanisms governing the metastatic organotropism of BC circulating tumor cells, helping to predict their preferential homing to specific sites. For example, bone and lung metastases show largely divergent transcriptome profiles, sharing only a limited set of genes, which underscores the tissue-specific requirements for survival of metastatic cells [5]. Given the rarity of intestinal BC metastasis, a tissue-specific gene signature for the intestine has not been fully elucidated. Interestingly, in a study of 23 primary breast tumors and their matched distant metastases, Schrijver et al. showed that miR-106b-5p is an independent predictor of gastrointestinal metastases [12].

Diagnosis of intestinal metastases is challenging. While PET scans have a high sensitivity for detecting distant metastases of malignant tumors, they can yield false-negative results due to variability in the metabolic activity of the metastases or if tracers are designed based on the receptor profile of the primary tumor [8]. In our patient, although PET-CT detected bone metastases, it did not reveal any hypermetabolic lesions in the gastrointestinal tract. In addition, GI endoscopy is often nondiagnostic, as the mucosa may be intact [13]. However, in our case, the diagnosis was successfully established through GI endoscopy, performed following the patient’s complaints of abdominal pain, which revealed an oozing, ulcerated lesion at the ileocecal valve; biopsy confirmed this to be metastatic ILC.

Histological confirmation is essential to differentiate metastasis from a primary gastrointestinal tumor, aided by immunohistochemical markers. GCDFP-15, mammaglobin, and GATA3 are relatively specific breast tumor markers, while ER/PR are also expressed [8]. In this case, high expression of GATA3 and ER supported the diagnosis of metastasis from the known breast tumor.

There is no consensus on the management of metastatic BC in the gastrointestinal tract. Chemotherapy and endocrine treatment are often implemented, while surgery is reserved for palliative relief if obstruction occurs. However, treatment decisions should consider potential changes in the receptor profile between the primary breast tumor and metastatic sites, a phenomenon known as receptor conversion [8]. In our case, the primary breast tumor was ER+/PR+ and treated with hormone therapy after mastectomy, while the metastatic lesion in the small intestine was ER+/PR-.

A study by Chen et al., which analyzed 390 BC cases with one or more distant metastatic sites, reported a higher PR expression loss (40.3%) compared to ER (18.3%) or HER2 conversion (13.7%) [14]. The loss of PR expression in metastases may result from hormone therapy selectively targeting ER+/PR+ cells, allowing ER+/PR− subpopulations to expand. This reflects clonal selection and tumor adaptation, potentially indicating resistance to endocrine therapy. Furthermore, a negative PR status in metastatic tumors, regardless of the primary tumor’s profile, was associated with a worse prognosis, compared to PR-positive tumors without conversion [14]. This underscores the complexity of managing metastatic BC as treatment is often guided by the primary tumor’s profile, necessitating adjustments in therapy.

At the time of the newly diagnosed intestinal involvement, our patient was already undergoing chemotherapy for bone marrow metastases. Due to disease recurrence and the emergence of intestinal metastasis, second-line treatment with letrozole and CDK4/6 inhibitors was initiated. CDK4/6 inhibitors have been introduced to overcome endocrine therapy resistance, a common challenge in hormone receptor-positive BC. By preventing the G1-to-S phase transition, these inhibitors effectively halt tumor cell proliferation and have been shown to improve overall survival, response rates, and recurrence-free survival, compared to hormone therapy alone [15,16]. This benefit has been observed not only in patients with localized disease but also in those with distant metastasis [15]. However, despite this treatment, our patient developed intestinal obstruction six months later, caused by a metastatic lesion, exhibiting receptor conversion and apparent treatment resistance. This highlights the evolving nature of metastatic BC, where shifts in receptor expression may impact treatment efficacy. Ongoing trials, such as AURORA, provide insight into the molecular landscape of metastatic BC, demonstrating that molecular changes are susceptible to existing targeted therapies, suggesting a potential benefit from molecular screening [17]. These findings emphasize the need for a personalized treatment approach that integrates molecular profiling and dynamic reassessment of tumor biology to refine therapeutic strategies.

## Conclusions

Metastatic involvement of the gastrointestinal tract in patients with ILC of the breast, although uncommon, represents a clinically significant and often underrecognized manifestation of metastatic disease. Histopathological and immunohistochemical analyses remain essential for accurate diagnosis, especially in distinguishing metastases from primary gastrointestinal malignancies. Surgical resection is palliative and aims to alleviate symptoms rather than prolong survival. In selected patients, however, surgery may significantly improve quality of life and facilitate continued systemic therapy. Additionally, the phenomenon of receptor conversion underscores the evolving tumor biology under therapeutic pressure and has important prognostic and therapeutic implications. As demonstrated in this case, discrepancies between the receptor profiles of primary and metastatic lesions may signal resistance to prior therapies and necessitate individualized adjustments in treatment. This case highlights the importance of dynamic reassessment of tumor biology during the course of metastatic disease and the importance of a multidisciplinary approach in the management of metastatic BC involving the gastrointestinal tract.
